# P-2225. Interactions between phage and host bacteria in immunized rabbit serum

**DOI:** 10.1093/ofid/ofae631.2379

**Published:** 2025-01-29

**Authors:** Sebastian Herren, Melissa J Karau, Christina Koscianski, Trace A Christensen, Robin Patel

**Affiliations:** Mayo Clinic, Rochester, Minnesota; Mayo Clinic, Rochester, Minnesota; Mayo Clinic, Rochester, Minnesota; Mayo Clinic, Rochester, Minnesota; Mayo Clinic, Rochester, Minnesota

## Abstract

**Background:**

Phage therapy is being evaluated to treat bacterial infections. Host-related factors that might abrogate phage efficacy remain incompletely characterized. Phage aggregation has been hypothesized to protect phages in harsh environments. Herein, effects of immunized rabbit sera on phage aggregation were assessed.

**Figure:**
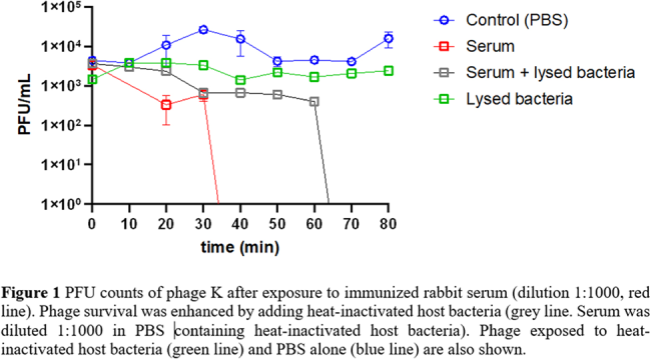

**Methods:**

Three New Zealand rabbits were immunized weekly for 3 weeks by intramuscular injection of phage K. Serum samples were collected weekly at times 0, 7, 14, 21 and 28. Development of an antibody response was confirmed by ELISA. Phage K was exposed to immunized serum with or without heat-inactivated host bacterium (*Staphylococcus aureus* ATCC 19685) as follows. 45 µl of phage K (10^10±1^ plaque forming units/mL [pfu/ml]) was mixed with 5 µl immunized serum diluted 1:1000 with or without heat-inactivated bacterial host in phosphate buffered saline (PBS). After incubation, exposed phage was visualized using transmission electron microscopy (TEM, JEOL) at 80kV.

**Figure:**
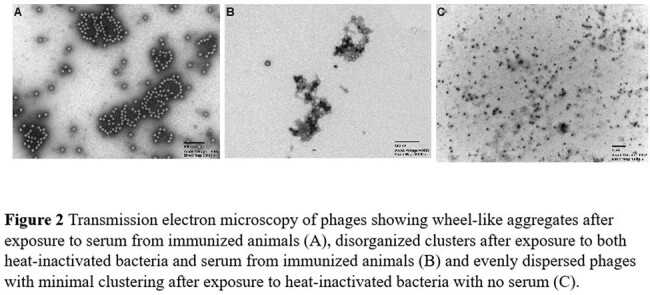

**Results:**

Phage K immunization elicited a strong IgG response. Exposure to immunized serum led to a decrease in viable phage over time, abrogated by adding heat-inactivated bacteria (Fig 1). TEM imaging showed phage clustering with exposure to immunized serum (Fig 2A); clusters appeared disorganized with concomitant exposure to heat-inactivated host bacteria (Fig 2B). Exposure of phage to heat-inactivated bacteria without serum yielded evenly distributed phage, with minimal clustering (Fig 2C).

**Conclusion:**

Phage K exposure elicited a neutralizing immune response associated with decreased phage survival in immunized serum, prolonged by adding heat-inactivated host bacteria. TEM imaging showed phage K clustering in different ways depending on the microenvironment encountered. Biological relevance and mechanistic underpinnings of these phenomena need further characterization.

**Disclosures:**

Robin Patel, MD, a patent on Bordetella pertussis/parapertussis PCR issued, a patent on a device/method for sonication with royalties paid by Samsung to Mayo Clinic, a: See above|MicuRx Pharmaceuticals and BIOFIRE: Grant/Research Support|PhAST, Day Zero Diagnostics, Abbott Laboratories, Sysmex, DEEPULL DIAGNOSTICS, S.L., Netflix, Oxford Nanopore Technologies and CARB-X: Advisor/Consultant|Up-to-Date and the Infectious Diseases Board Review Course.: Honoraria

